# From Waste to Value: Fruit Biofillers in Biodegradable Composite Materials

**DOI:** 10.3390/biomimetics11040274

**Published:** 2026-04-15

**Authors:** Smaro Kyroglou, Antigoni G. Margellou, Konstantinos S. Triantafyllidis, Patroklos Vareltzis

**Affiliations:** 1 Laboratory of Food and Agricultural Industries Technologies, Chemical Engineering Department, Aristotle University of Thessaloniki, 54124 Thessaloniki, Greece; kyrosmar@cheng.auth.gr; 2 Department of Chemistry, Aristotle University of Thessaloniki, 54124 Thessaloniki, Greece; amargel@chem.auth.gr; 3 Chemistry Department, King Fahd University of Petroleum & Minerals, Dhahran 31261, Saudi Arabia; k.triantafyllidis@kfupm.edu.sa; 4 Interdisciplinary Research Center for Refining and Advanced Chemicals, King Fahd University of Petroleum & Minerals, Dhahran 31261, Saudi Arabia

**Keywords:** fruit waste valorization, biodegradable composites, thermocompression processing, binderless biocomposites, mechanical properties

## Abstract

This study addresses the urgent need for sustainable alternatives to single-use plastics by developing biodegradable composites from peach and apple processing waste employing hot compression molding. Utilizing a definitive screening design, the impact of the process variables, including recipe composition, grinding size, pressure, temperature, and holding time, on the physical (including water resistance) and mechanical properties of the composites was systematically evaluated. Physicochemical and thermal analyses of the dried by-products indicated that processing temperatures below 150 °C prevent the degradation of lignocellulosic constituents. The results demonstrated that increasing both the molding pressure and holding time decreased the composite thickness, while enhancing the stiffness and flexural strength, with modulus of elasticity values exceeding 1000 MPa under optimal conditions. Higher molding temperatures reduced water absorption and diffusivity, particularly in lignin-rich composites, by promoting lignin softening and particle consolidation, resulting in denser structures with limited moisture transport. Biodegradability was assessed through soil burial tests over 200 days, revealing a weight loss ranging from 54.2% to 90.7% among samples, with apple-based composites exhibiting greater degradation compared to peach-based ones. Overall, the study highlights the development of a “green composite” formulation inspired by biomimetic principles, exploiting the natural self-bonding capacity of lignocellulosic biomass, where two different-in-composition biowastes are combined to produce a plastic-free composite material with possible applications in the foodservice industry.

## 1. Introduction

The estimated annual use of single-use plastic items (e.g., straws) reaches approximately 25.3 billion units in Europe. Plastic products are preferred because of their low price and high durability [1]. However, the massive volume of plastic derivatives discarded daily poses severe environmental threats to marine and terrestrial ecosystems. The European Union has announced a ban on single-use plastics by 2030 [2]. Specifically, the European framework is primarily governed by the Single-Use Plastics (SUP) Directive [3], which aims to reduce marine litter and plastic production. The directive bans single-use plastic cutlery (forks, knives, spoons, chopsticks), plates, straws, stirrers, and balloon sticks and promotes the use of sustainable, non-plastic alternatives [3]. To reduce plastic waste and ensure compliance with European Union regulations, there has been a notable transition by both industry and consumers toward biodegradable foodservice products. These alternatives are designed to undergo complete microbial degradation into water, carbon dioxide, and biomass under controlled conditions. The global biodegradable tableware market is projected to increase from USD 39.82 million in 2024 to USD 73.43 million by 2032, corresponding to a compound annual growth rate (CAGR) of 7.95% during the forecast period [4].

Available biodegradable products are primarily based on natural-origin polylactic acid, paper, cellulose, cellulose–lignin composites, and bacterial cellulose–alginate blends. However, these materials exhibit several limitations, including high production cost, low resistance to heat and water, and rapid softening within only a few minutes [5,6,7,8]. The utilization of agricultural waste and residues as raw materials represents a promising strategy that not only reduces the solid waste, typically disposed of in landfills, rivers, and marine environments, but also enables the valorization of valuable constituents such as polysaccharides and phenolic compounds. Industrial fruit by-products include leaves, peels, pits, stems, seeds, spoiled fruits, and pulp, which are rich in starch, pectin and cellulose. Other properties of agricultural waste such as availability, low cost, abundance, environmental compatibility and physicochemical profile render it an excellent candidate for the substitution of plastic and its derivatives.

Recent studies have examined the use of orange [9], banana [10], mango [11], watermelon [12], apple and durian peel [13], and pineapple [14] for the production of biodegradable cups, straws and films. Other researchers studied the valorization of grape pomace and tomato processing residues for packaging applications [15,16,17]. Similarly, binderless composites have been developed from agricultural residues such as sugarcane bagasse [18], rice straw [19], vineyard pruning [20], and bamboo [21] by exploiting the self-bonding capacity of lignocellulosic materials under hot pressing. The main processing technologies are hot compression molding using a hydraulic press machine and extrusion. Hot compression molding involves placing the fruit-based biopolymer mixture into a heated mold and applying controlled pressure for a defined period. This step softens and consolidates the matrix, enhances interfacial bonding between components, and produces dense, dimensionally stable biocomposites. In contrast, extrusion processes the same formulation by conveying it through a heated barrel with rotating screws. The combined effects of shear, pressure, and elevated temperature induce the plasticization and homogenization of the material, which is subsequently forced through a die to produce continuous strands or sheet-like structures. Moreover, the integration of extrusion followed by hot-press molding, applied to rice bran-based matrices reinforced with nanoclay (montmorillonite), resulted in bioplastics with substantially improved mechanical performance. The storage modulus increased by 35%, tensile strength by 15%, and elongation at break by 30%, underscoring the synergistic effects of the technologies’ combination [22]. This finding has also been confirmed by other researchers, who produced biocomposites from extruded apple pomace combined with potato starch [23].

This work introduces an innovative approach to biodegradable composite fabrication by valorizing peach and apple industrial residues without the need for added binders, plasticizers, or synthetic reinforcement, distinguishing it from most existing fruit waste biocomposites that rely on starch matrices, PLA blends, or nanoparticle fillers. From a biomimetics perspective, this study utilizes natural plant structures, in which lignocellulosic components provide mechanical strength and cohesion without added binders. Processing under the appropriate temperature and pressure converts lignin to a thermoplastic matrix. This natural self-bonding mechanism aligns with the principles of biomimetics by utilizing natural structures for the production of engineered materials.

This study addresses gaps identified in recent reviews, which highlight the intrinsic variability in agricultural residues (affecting processing performance and product consistency) by systematically investigating the influence of two compositionally different biowastes (peach and apple) on thermocompression processing, with the aim of developing a fully bio-based composite capable of replacing bioplastics and synthetic reinforcements [24]. Through the implementation of a definitive screening design, the study provides a systematic and comprehensive evaluation to date of how processing conditions (recipe, particle size, temperature, and holding time) govern the density, water transport behavior, mechanical performance, and biodegradation evolution of lignocellulosic composites without binders. This work advances the understanding of structure–process–property relationships in waste-derived lignocellulosic materials, particularly the role of natural extractives, lignin content, and pressing-induced microstructural densification in determining diffusivity and biodegradation. From an industrial perspective, the method offers a low-cost and scalable pathway for converting abundant fruit-processing residues into functional materials suitable for eco-friendly packaging, single-use foodservice items, and disposable consumer goods, supporting the transition toward circular bioeconomy models and compliance with upcoming single-use plastic restrictions.

## 2. Materials and Methods

### 2.1. Raw Materials and Reagents

Fruit processing by-products derived from peaches and apples were supplied by a local industry (Kronos S.A., Skidra, Greece). Τhe waste material consisted of fruit peels, with an initial moisture content in the range of 70–75%.

### 2.2. Sample Preparation

The peach and apple residues were sun-dried during the summer period until constant weight was achieved. Subsequently, the dried materials were milled (Magico 1 EM 50, AMA, Reggio Emilia, Italy) using sieves of three different mesh sizes (7 mm, 3.9 mm and 0.8 mm), vacuum-sealed (MULTIVAC, C200, Wolfertschwenden, Germany), and stored until further use.

### 2.3. Characterization of Dried By-Products

#### 2.3.1. Determination of Physicochemical Properties

The moisture and ash content of peach and apple by-products were gravimetrically determined at 105 °C till constant weight according to AOAC 925.10 and at 575 °C (Digital muffle furnace, FX-05, Witeg, Wertheim, Germany) for 3 h according to AOAC 936.03 respectively [25]. Moreover, the bulk density of the dried materials was calculated by dividing the mass of the sample by its volume.

#### 2.3.2. Determination of Lignocellulosic Composition

The determination of lignocellulosic composition was determined according to the NREL/TP-510-42619 and NREL/TP-510-42618 protocols. Prior to the analysis of structural components, the extractives content was determined by exhaustive Soxhlet extraction first with ultrapure water and then with ethanol according to the NREL/TP-510-42619 protocol [26]. The analysis of water extractives was performed via High-Performance Liquid Chromatography (HPLC, LC-20AD, Shimadzu, Tokyo, Japan), using a refractive index detector (RID-6A, Shimadzu) and an oven (CTO-20A, Shimadzu).

The structural carbohydrates and the lignin content were determined after double-acid hydrolysis, according to the protocol NREL/TP-510-42618 [27]. According to this procedure, in the first step, the biomass is treated with 72% H_2_SO_4_ at 25–30 °C for 1 h and subsequently an adequate amount of water was added to obtain 4% H_2_SO_4_ and the mixture was treated at 121 °C for 1 h. Afterwards, filtration was performed to separate the carbohydrates and the acid-soluble lignin in the liquid fraction from the acid-insoluble lignin in the solid. The monomeric sugars (glucose, xylose, galactose, mannose, and arabinose) were determined after neutralization with CaCO_3_, via HPLC (LC-20AD, Shimadzu, Tokyo, Japan), using a refractive index detector (RID-6A, Shimadzu) and an oven (CTO-20A, Shimadzu), equipped with an SP-0810 Sugar Column (Shodex, Tokyo, Japan), with ultrapure H_2_O as eluent at 80 °C and a flow rate of 0.6 mL/min. Acid-soluble lignin was determined by UV spectroscopy, measuring the absorbance at 240 nm while the acid-insoluble lignin was determined gravimetrically, after drying the recovered solid at 105 °C.

#### 2.3.3. Thermal Properties (TGA, DTG)

Τhermogravimetric analysis (TGA/DTG) was performed on a thermal analyzer (STA 449 F5, Netzsch, Selb, Germany). About 10 mg of the sample was heated under N_2_ flow and a constant heating rate of 10 °C/min in the temperature range of 25–950 °C.

#### 2.3.4. X-Ray Diffraction Analysis (XRD)

XRD was performed using a Bruker D8 Advance X-ray diffractometer (Bruker AXS GmbH, Karlsruhe, Germany) equipped with a filtered Cu radiation source (λ = 1.54060 Å) at the operating voltage and current of 40 kV and 40 mA, respectively, and a LynxEye detector. The samples processed as described above were put in the XRD pan where the room temperature was maintained. The resulting spectra were analyzed using “Diffrac. Eva” software v7 (Bruker AXS GmbH, Karlsruhe, Germany).

### 2.4. Preparation and Characterization of Biodegradable Composites

#### 2.4.1. Production of Composites

The formation of biodegradable composites was carried out using a hydraulic press machine (RYJ-600Z2, TMAXCN, Xiamen, China) and a stainless steel rectangular-shaped mold (Length: 12 cm, Width: 2.85 cm). Preliminary experiments were conducted to determine the required quantity of dried materials, as well as to identify the upper and lower limits of the operational parameters of the hydraulic press. Based on these results, a 3-level definitive screening design involving five factors (recipe, grinding size, pressure, temperature and holding time) was set up using Minitab ^®^ 21 (Minitab, Ltd., Coventry, UK). The recipe ranged from 0% to 100% peach waste, grinding size from 0.8 mm to 7 mm, pressure from 2 to 24 T/m^2^, temperature from 50 °C to 150 °C and holding time from 4 to 12 min. Based on the experimental design, 13 distinct experimental conditions were defined, each performed in triplicate, resulting in a total of 39 experiments. The experimental design, selected for the production of the biodegradable composites, is presented in Appendix A (Table A1). Each experimental condition was assigned a numerical code (1–13), corresponding to a unique combination of processing parameters, while individual replicates were denoted by letters (A, B, and C). Thus, sample codes such as 1A, 1B, and 1C represent the three replicates of experimental condition 1. The values reported for samples 1–13 correspond to the average of their respective three replicates.

#### 2.4.2. Thickness and Density

The thickness of the composites was measured with a digital caliper (1114, INSIZE, Suzhou New District, China). Thickness measurements were taken at ten random points and the mean value was used to determine the water vapor permeability and flexural properties. The density of the samples was determined by dividing their mass by their volume, where the volume was calculated assuming the geometry of a rectangular solid, as given by Equation (1):

(1) Volume=Length×Width×Thickness 

#### 2.4.3. Water Absorption Capacity

Water absorption was tested by immersing 2 cm × 2 cm specimens in 25 mL of ultrapure water at 25 °C. Before soaking, the samples were oven-dried at 105 °C until constant weight to ensure a uniform initial condition. The initial weight (W_0_) was then measured. The samples were taken out at various time intervals (1, 3, 5, 10, 15, 30, 45, 60 and 75 min), blotted with filter paper, and weighed (W_t_). The experiment was conducted in triplicates. Water absorption was calculated by using Equation (2):

(2) $$Water\;absorption\;\%=\frac{W_{t}-W_{0}}{W_{0}}\times 100$$

Furthermore, the effective diffusion coefficient was determined from sorption data using the solution of Fick’s second law for a rectangular slab of known thickness. Moisture uptake was measured at predetermined intervals and the diffusion coefficient was calculated by fitting the data to the model for unsteady-state diffusion in a slab geometry. Equations (3)–(5) were used for the calculations [28]:

(3) $$M_{t}=M_{\infty }\left\{1-\frac{8}{\pi^{2}}\overset{\infty }{\underset{n=0}{\sum }}\frac{1}{{\left(2n+1\right)}^{2}}exp\left[-\frac{{\left(2n+1\right)}^{2}\pi^{2}D_{eff}\;t}{h^{2}}\right]\;\right\}$$

For short times (initial linear region) and M_t_/M_∞_ < 0.6, the Equation (3) can be simplified to the following:

(4) $$M_{t}=\frac{4M_{\infty }}{h}\sqrt{\frac{Dt}{\pi }}$$

Τhe effective diffusivity is obtained from the slope of a plot of $\frac{M_{t}}{M_{\infty }}$ versus $\sqrt{t}$:

(5) $$D_{eff}=\frac{\pi \times slope^{2}\times h^{2}}{16}$$

#### 2.4.4. Flexural Properties

Flexural properties were measured using an Instron 3344 dynamometer (Instron, Norwood, MA, USA), equipped with a 2 kN load cell (accuracy class: 1–100% of load cell capacity). The measurements were carried out using the 3-point bending method in accordance with ASTM D790 [29], at a crosshead speed of 50 mm/min. At least 5 specimens of rectangular parallelepipedal shape were used for each sample (Figures S1 and S2).

#### 2.4.5. Biodegradability Test

The most common actual standard soil biodegradation methods are ASTM D5988-18 [30] and ISO 17556:2019 [31], based on the oxygen demand or the amount of carbon dioxide evolved. In the present study, a soil burial test was performed in accordance with the weight-loss method described by Kumar et al. (2010) with slight modifications [32]. To perform this test, samples measuring 2 × 2 cm were weighed (W_0_). Each sample was placed inside a polyester mesh and tied with thread in the form of a pouch. The pouches were buried at a depth of 5 cm in the soil at ambient temperature. The soil had a pH of 7.0, and moisture content was maintained at 50–60% by sprinkling water every 2 days. After 10 days, the samples were collected and washed with distilled water, then dried in an oven at 60 °C for 2 h and weighed again (W_t_). Βiodegradability was recorded as weight loss at fixed time intervals with a constant frequency of 10 days. The weight loss of the samples was calculated by using Equation (6):

(6) $$Weight\;loss\;\left(\%\right)=\frac{W_{0}-W_{t}}{W_{0}}\;$$

### 2.5. Statistical Analysis

The design of experiments (DOEs) was established using a three-level definitive screening design (DSD) with five factors. The DSD analysis was employed to evaluate the main effects (time, pressure, recipe, grinding size and temperature) on the response variables (i.e., thickness, density, diffusion coefficient, flexural strength and biodegradability) according to the F- and *p*-values of the corresponding ANOVA tables. Individual responses’ values were analyzed by ANOVA (one-way analysis of variance) with Tukey’s test to compare means. Significance was reported at the *p* < 0.05 level. Data are presented as mean values ± standard deviation (SD) obtained from three independent analyses (*n* = 3). For the mechanical properties (modulus of elasticity and flexural strength), five samples were tested. Minitab^®^ 21 (Minitab, Ltd., Coventry, UK) statistical software was used for the statistical analysis.

## 3. Results

### 3.1. Physicochemical Profile of Dried By-Products

The peach and apple dried by-products were characterized for their moisture, ash content and density. The results are presented in Table 1.

### 3.2. Lignocellulosic Analysis of Biomass

**Figure A1:**
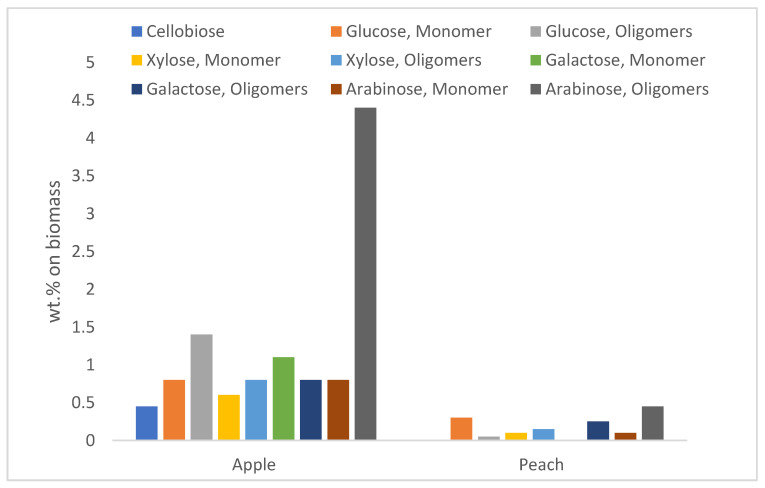
Analysis of water extractives.

The chemical composition of peach and apple dried wastes, including the structural (cellulose, hemicellulose and lignin content) and the non-structural components (ash and extractives), is shown in Table 2. Both initial feedstocks (prior to the removal of extractives) exhibit a high glucan content ranging from 17.7 to 20.6 wt.%. Peach wastes are enriched in xylan (11.4 wt.%) compared to apple wastes, which exhibit a significantly lower xylan content 5.2 wt.%. Furthermore, peach wastes exhibit a higher mannan content (3.9 wt.%). On the other hand, the galactan content is slightly higher (5.5 wt.%) in apple wastes than peach wastes (4.2 wt.%). The main biopolymer in both samples is lignin, with a content of 49.8 wt.% for peach wastes and 37.4 wt.% for apple wastes. Regarding the non-structural components of wastes, apple-derived wastes exhibit a remarkably higher amount of extractives, 61.9 wt.%, compared to peach-derived wastes with 28.9 wt.% total extractives content. A significant difference between the two types of wastes is the nature of extractives. Apple wastes exhibit a higher amount of water-extracted compounds (45.5 wt.%), while peach wastes exhibit mainly compounds extracted in ethanol (20.4 wt.%). As can be observed in Figure A1 (Appendix A), the analysis of water extractives revealed the existence of C_5_ and C_6_ monomeric and oligomeric sugars. Among peach- and apple-derived compounds, the latter exhibit a higher amount of extractives and especially arabinose oligomers. Regarding the analysis of extractives-free samples, the cellulose content expressed as glucan ranges from 30.3 to 33.2 wt.% and is higher for the apple powder. Hemicellulose components are in the range of 21.3–24.4 wt.% with peach waste exhibiting the highest content of hemicellulose sugars, mainly xylan. The lignin content is 42.5 and 43.7 wt.% for apple and peach, respectively. The decrease in lignin content after extractives removal in peach wastes indicates that are partially derived from non-structural components. Similarly, the decrease in acetyl units and ash contents is attributed to non-structural components.

### 3.3. Thermogravimetric Analysis

**Figure 1:**
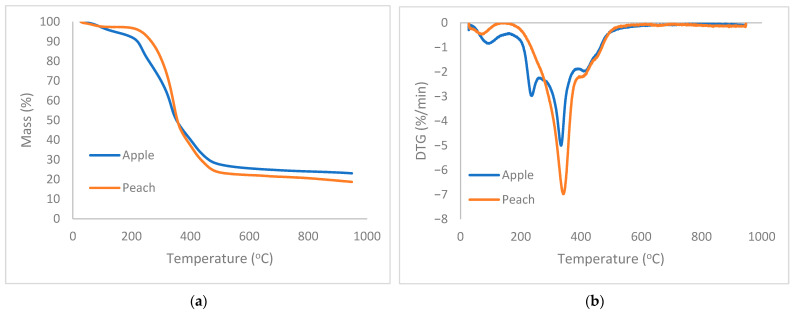
(**a**) TGA and (**b**) DTG curves of peach and apple by-products.

The thermogravimetric analysis (TGA) and derivative thermogravimetric (DTG) profiles of apple and peach by-products, used as raw materials for biodegradable composites, are presented in Figure 1a,b. The TGA curves reveal three distinct stages of mass loss for both materials. The initial weight loss observed below 150 °C corresponds to moisture evaporation. A major decomposition phase occurs between approximately 200 °C and 400 °C, attributed to the degradation of hemicellulose, cellulose, and partial lignin components. Beyond 400 °C, the mass stabilizes, indicating the formation of residual char. Comparatively, peach exhibits a slightly higher mass loss across the temperature range, suggesting lower thermal stability relative to apple.

The DTG curves show a small peak around 200–250 °C associated with hemicellulose breakdown. This feature is clearer for apple. The second decomposition region is distinguished by the primary peak between 300 °C and 350 °C for both materials, corresponding to the most rapid thermal decomposition associated with cellulose degradation. Notably, peach displays a sharper and higher DTG peak at the main decomposition stage, indicating a faster degradation rate in this temperature range. Lignin decomposes over a broad temperature range from 200 °C to 500 °C. Therefore, the broad tailing after the main peak can be attributed to lignin decomposition. These thermal profiles set the upper limit of the processing temperatures for composite fabrication using the hydraulic press machine, for the specific parameters and fruit types investigated in this study. The upper temperature of the definitive screening design (Appendix A, Table A1) was 150 °C, significantly below 200 °C, to avoid thermal degradation of the raw materials.

### 3.4. XRD Analysis of Dried By-Products

**Figure 2:**
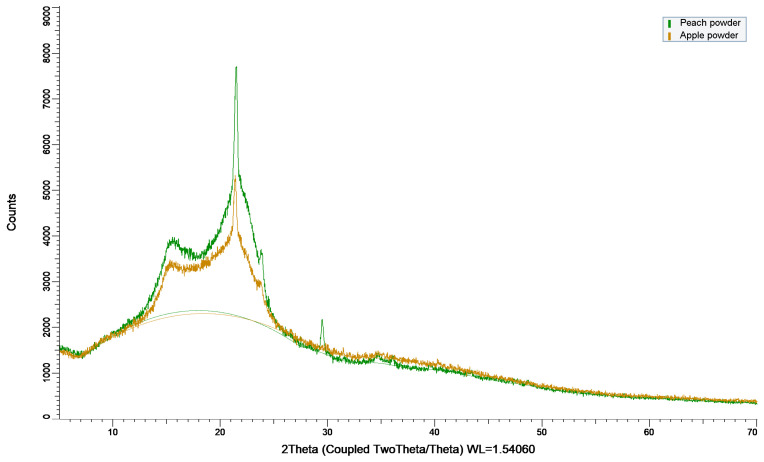
XRD curves of peach and apple powder.

X-ray diffraction analysis revealed that both apple peel and peach peel powders exhibit semi-crystalline structures dominated by cellulose I, with a characteristic diffraction peak at 2θ ≈ 21–22°. Peach peel powder displayed a markedly higher peak intensity and sharper diffraction features, indicating a higher degree of cellulose crystallinity compared to apple peel powder (Figure 2). Specifically, the crystallinity index of peach and apple peel was 48.5% and 36.3% respectively. The latter showed a stronger amorphous contribution, attributable to a higher pectin, hemicellulose, and lignin content.

### 3.5. Characterization of Biocomposites

#### 3.5.1. Physical and Water Absorption Properties of Biocomposites

The thickness, density and moisture diffusion coefficients of the composites are presented in Table 3. Significant differences have been recorded for the thickness among the composites. The highest value was measured for sample 10, whereas the lowest one was for sample 11. The density values ranged from 0.858 to 1.544 g/cm^3^.

**Figure 3:**
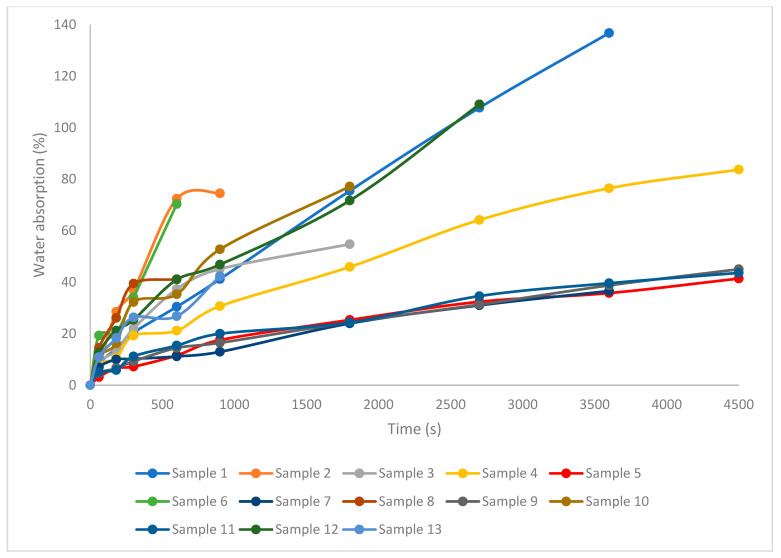
Water absorption of biocomposites.

Figure 3 presents the evolution of water absorption over time. At 300 s of immersion, sample 8 exhibited the highest water absorption (39.46%), whereas sample 5 showed the lowest uptake (7.23%). The moisture diffusion coefficient values were between 1.54 × 10^−11^ and 44.13 × 10^−11^ m^2^/s.

#### 3.5.2. XRD Analysis of Biocomposites

**Figure 4:**
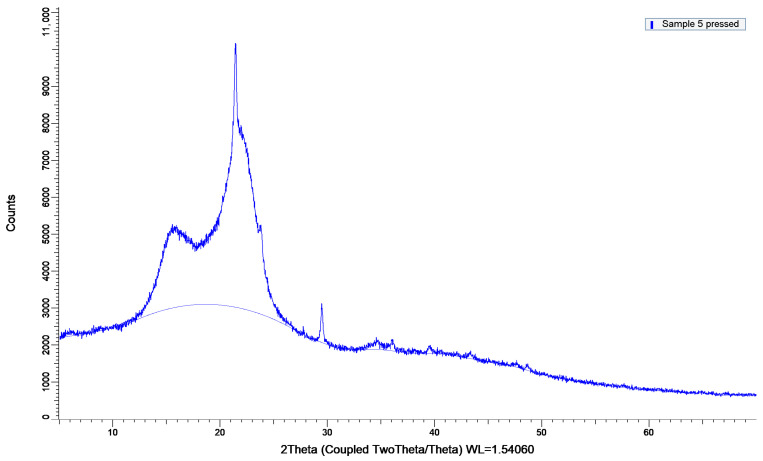
XRD curve of sample 5.

**Figure 5:**
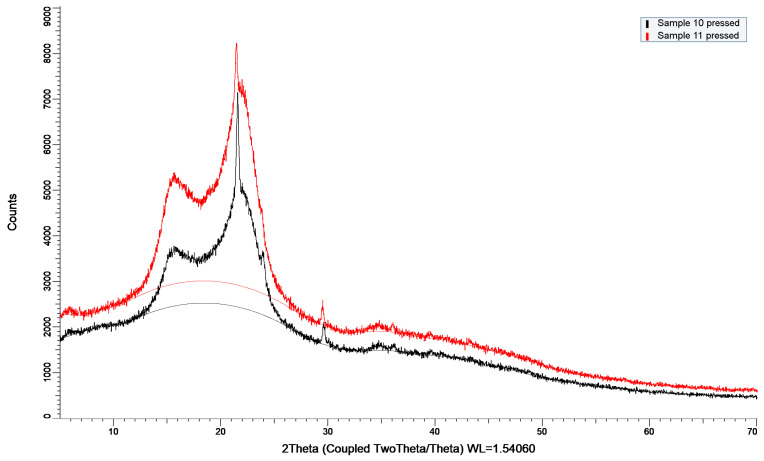
XRD curves of sample 10 and 11.

Since the main effects plot (Figure A5) showed that temperature is the main factor affecting flexural strength, composites 5, 10 and 11 were chosen for further investigation. Figure 4 and Figure 5 show the XRD patterns of those composites. Two 50/50 composites were molded at the lowest and highest temperatures, and one 100% peach composite was processed at a high temperature. The calculated crystallinity indices for these materials were 53.4% for composite 5, 43.5% for composite 10, and 54.3% for composite 11. Sample 5, which consisted of 100% peach peel, exhibited a slightly higher crystallinity index than peach peel powder, likely due to the high processing temperature. Sample 10, produced from a 50:50 mixture of peach peel and apple peel under mild processing conditions (2 T/m^2^, 50 °C), showed a crystallinity index of 43.5%, which is close to the weighted average of the two individual powders. In contrast, maintaining the same composition while applying a combination of high pressure and high temperature resulted in a marked increase in the crystallinity index (54.3%) of sample 11.

#### 3.5.3. Mechanical Bending Properties

**Figure 6:**
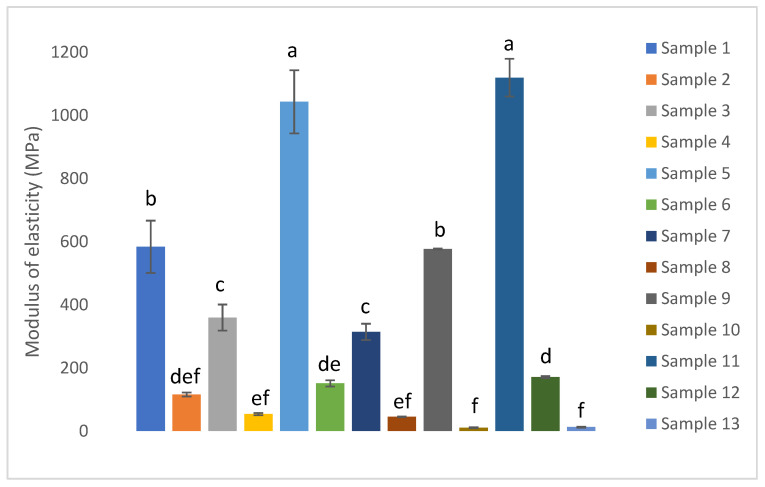
Modulus of elasticity of the composites.

**Figure 7:**
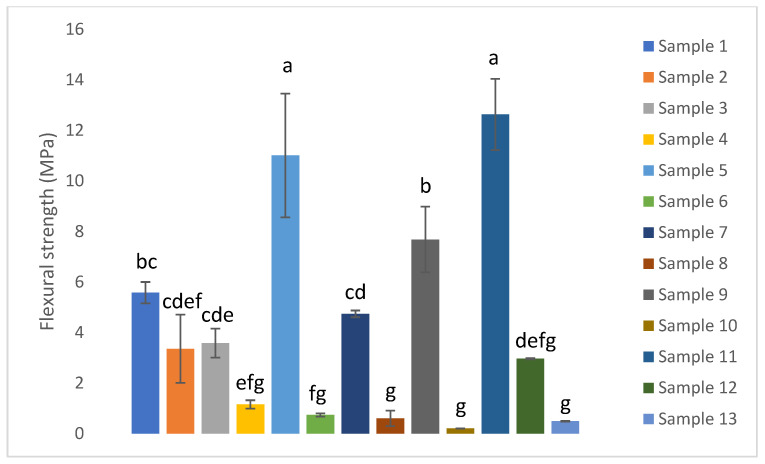
Flexural strength of the composites.

The modulus of elasticity and flexural strength for samples 1 to 13 are presented in Figure 6 and Figure 7 respectively.

The modulus of elasticity of the composites ranged from 10.7 to 1119.5 MPa, while their flexural strength varied between 0.2 and 12.6 MPa. Sample 11 exhibited the highest values for both properties, indicating superior stiffness among the tested composites. Moreover, the modulus of elasticity and the flexural strength followed parallel trends across the series, such that samples with a higher modulus of elasticity also displayed a higher flexural strength.

#### 3.5.4. Biodegradability Analysis

**Figure 8:**
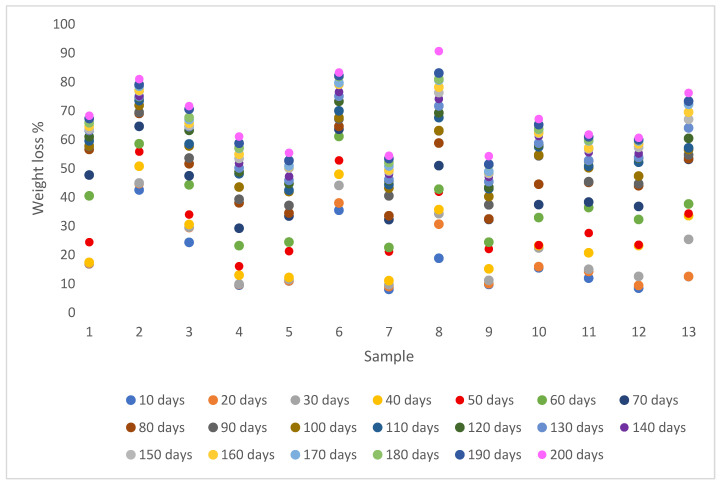
Weight loss of composites from 10 to 200 days.

The percentage of weight loss for all the samples buried in soil from 10 to 200 days is presented in Figure 8. The percentage of degradation gradually increased over time, while the highest weight loss was 90.7% for sample 8 in 200 days. On the other hand, sample 9 had the lowest biodegradation equal to 54.2% in 200 days.

## 4. Discussion

### 4.1. Physicochemical Properties of Dried By-Products

Peach by-product consists of 20.6% cellulose, 19.7% hemicellulose and 49.8% lignin. According to Toushik et al. (2017), in the case of peach pomace, cellulose accounts for 28.7–30.0%, hemicellulose for 18.6–20.0%, and lignin for 5.35–6.0% [33]. The ash content was 2.5%, which is lower than previously reported values ranging from 4.11% to 4.3% [34].

Furthermore, apple by-product consists of 17.7% cellulose, 12.8% hemicellulose and 37.4% lignin. These findings are consistent with other studies, where apple pomace composition was reported (7–44% cellulose, 4–24% hemicellulose, and 15–23% lignin) [35,36,37]. The ash content for apple peel was 3.2%. Velciov et al. (2022) found that the ash content of apple peels from different batches varied from 1.8 to 4.8% [38]. The lignin content was significantly higher for peach and apple waste compared to that reported by other researchers. This could be attributed to the quality of fruit waste, because peels from fruits at advanced ripening (main raw material of fruit processing industries) could exhibit increased pericarp lignification as a protective response [39].

The TGA and DTG results showed that the major decomposition of apple and peach biomass components (hemicellulose, cellulose, lignin) occurs above ~200 °C, with peak degradation rates around 300–350 °C. Processing below 150 °C avoids these degradation temperatures, preserving fiber polymer chains and crystalline cellulose structures. This prevents the weakening or embrittlement of fibers, which directly influences mechanical properties like the modulus of elasticity and flexural strength. Furthermore, the moisture evaporation below 150 °C reinforced the interfacial bonding during the compression. High pressure also significantly affected the contact of the particles, eliminating the voids and providing sufficient adhesion. Similar results were observed by Merino and Athanassiou (2023), who showed that excessive heat in carrot pomace composites led to diminished mechanical properties due to fiber breakdown [40]. Lima et al. (2021) highlighted the effect of the processing conditions on the formation of PLA biocomposites with the addition of kernel from mango seeds, which started to decompose during the extrusion because of the high amount of sugars and carbohydrates [11].

### 4.2. Properties of Biocomposites

**Figure A2:**
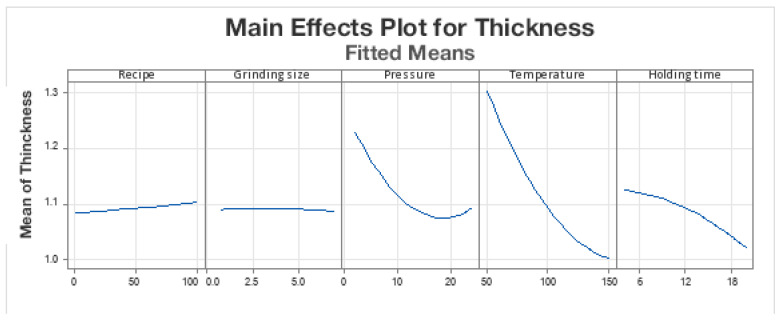
Main effects plot for thickness.

The thickness of the biocomposites was influenced by the pressure, temperature and holding time (*p* < 0.05, Table S1, Figure A2). The thinnest sample (Sample 11) was produced by applying the highest pressure and holding time. The combination led to increased compaction and consequently the thickness was reduced by pushing particles and fibers closer together. Moreover, sample 10 was characterized by the highest thickness and the lowest density among the other samples. The pressure and holding time were set to the lower limit, causing insufficient compaction, more voids inside the sample and decreasing the mass per volume.

**Figure A3:**
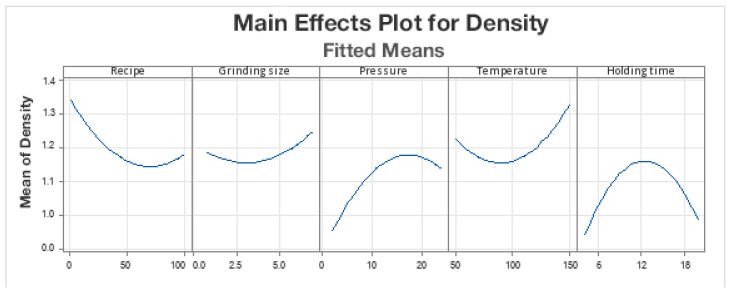
Main effects plot for density.

Reported densities of biodegradable composites derived from fruit and vegetable waste typically range from 0.5 to 1.5 g/cm^3^, depending on factors such as the type of by-product, the matrix material, filler content, and processing conditions. Cavailles et al. (2024) highlighted the influence of the thermocompression conditions on the properties of bio-based materials from sugarcane bagasse. Specifically, the material density increased with the molding pressure until a maximum density of around 1.460 g/cm^3^ was reached at ≥68 MPa [41]. De Melo Barbosa et al. (2022) observed densities from 0.8 to 1 g/cm^3^ in polymeric composites blended with açaí seed residue, noting increases in density correlated to a smaller particle size [42]. Similarly, Harahap et al. (2025) documented densities from 0.6034 to 0.6846 g/cm^3^ for biodegradable pots produced from empty fruit bunches, highlighting the influence of the composition [43]. In addition, Nukala et al. (2022) reported that the density of biodegradable composites made from polycaprolactone was reduced with the addition of bamboo powder [44]. These findings align with the density values obtained in the present study ranging from 0.858 to 1.544 g/cm^3^, underscoring the critical role of the recipe and processing parameters, such as the pressure, temperature, holding time, and particle size (*p* < 0.05, Table S2, Figure A3), in controlling the final composite microstructure and density, which in turn impact the mechanical strength and durability.

**Figure A4:**
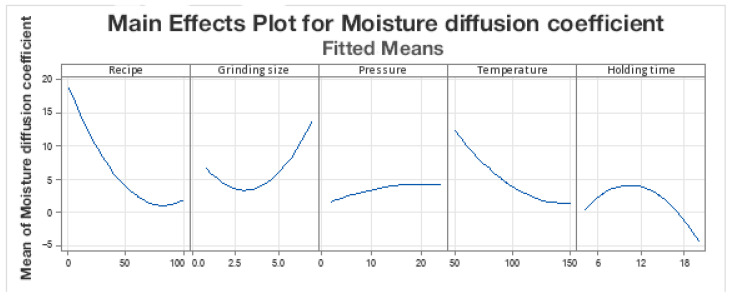
Main effects plot for moisture diffusion coefficient.

**Figure A5:**
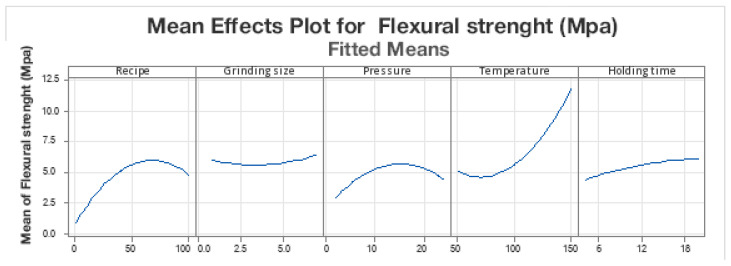
Main effects plot for flexural strength.

Water absorption and effective diffusivity were affected by the synergistic action of composite formulation and pressing temperature (*p* < 0,05, Table S3, Figure A4). Increasing the molding temperature from 50 °C to 150 °C decreased water absorption and the effective diffusivity, with the most pronounced reductions observed in the 100% peach and mixed apple/peach systems, which contain higher lignin contents than the 100% apple formulations (*p* < 0.05, Table S4). In these lignin-richer systems, a higher temperature promotes the softening of lignin and other amorphous constituents, enhancing particle coalescence and closure of transport pathways, whereas at 50 °C, lignin remains predominantly glassy and less mobile, preserving porosity and facilitating water uptake. The composites are lignocellulosic materials without binders and the main cause of dimensional instability was the hydrophilic character of hemicellulose [41,45]. A similar explanation was given by other researchers, supporting that the natural fibers mainly consist of cellulose, hemicellulose, lignin, and other natural proteins, which have high water-retaining capacities [46], and hence, more water-absorbing pores can be formed [44].

The effect of the temperature was further modulated by the particle size (Figure A4): coarse powders (7 mm) pressed at 50 °C exhibited relatively high diffusivities (44.13 × 10^−11^ m^2^/s for sample 8), while the same coarse systems processed at 150 °C showed markedly lower values (1.54 × 10^−11^ m^2^/s for sample 11), indicating that an elevated temperature is particularly critical for mobilizing lignin across larger particle interfaces. These observations align with recent reports on hot-pressed lignocellulosic and fruit pomace materials, where moisture diffusivity decreases when the processing conditions promote lignin softening, interdiffusion, and pore closure, leading to more compact, less permeable microstructures [47,48]. Similar values of the moisture diffusion coefficient were mentioned by Gozdecki et al. (2025), who produced composites using biopolymer and waste coffee husks with an effective diffusivity coefficient from 2.69 × 10^−11^ m^2^/s for 10 wt.% coffee husk to 3.03 × 10^−11^ m^2^/s for 50 wt.% coffee husk [49].

The modulus of elasticity and flexural strength of biodegradable composites ranged from 10.7 to 1119.5 MPa and 0.2 to 12.6 MPa respectively. Other researchers reported an elastic modulus for such biocomposites fluctuating from a few hundred MPa up to over 1400 MPa, with flexural strengths spanning approximately 5 to 30 MPa. Specifically, Kumar et al. (2022) achieved a tensile modulus of 851.33 MPa and flexural strength of 27.59 MPa by reinforcing biocomposites with 30 wt% pineapple leaf fibers [14]. Similarly, Chaudhary et al. (2025) reported a flexural strength ranging from 30 to 85 MPa and elastic modulus from 563.67 to 900 MPa in starch-based biocomposites incorporating fruit biofibers, emphasizing the critical role of the plasticizer content and molding temperature on mechanical behavior [50]. Moreover, Silva et al. (2025) developed chestnut shell-based composites, and the optimal formulation achieved a flexural strength of 9.00 MPa and a flexural modulus of 950 MPa, revealing how the composition and processing parameters influence mechanical performance [51]. These values align well with the mechanical properties observed in this study, particularly for the best-performing samples whose modulus of elasticity exceeded 1000 MPa and flexural strengths reached above 10 MPa.

The structural changes inferred from XRD correlate strongly with the mechanical performance of the composites. The 50/50 peach–apple peel composite processed at 24 T and 150 °C exhibited the highest flexural strength, resulting from the combined effects of an increased crystallinity index and pronounced densification. A higher crystallinity index contributes to improved stiffness and load-bearing capacity through the reinforcing role of crystalline cellulose domains, as reported by Poletto et al. (2014) [52]. Moreover, Chen et al. (2020) observed that the crystallinity of wood biocomposites increased with the increase in the hot-pressing pressure, reflecting that a considerable amount of crystalline cellulose regenerated during the hot-pressing process [53]. However, crystallinity alone is insufficient to explain the observed mechanical enhancement. The significant reduction in composite thickness under high-pressure/high-temperature processing indicates a lower porosity and improved particle to particle contact, which are critical for efficient stress transfer during flexural loading. This is supported by the lower flexural strength of the 100% peach peel composite pressed at 2 T and 150 °C despite its high crystallinity index. Overall, the results demonstrate that the superior mechanical performance arises from the synergistic interaction between the crystallinity, densification, and optimized composite microstructure.

**Figure A6:**
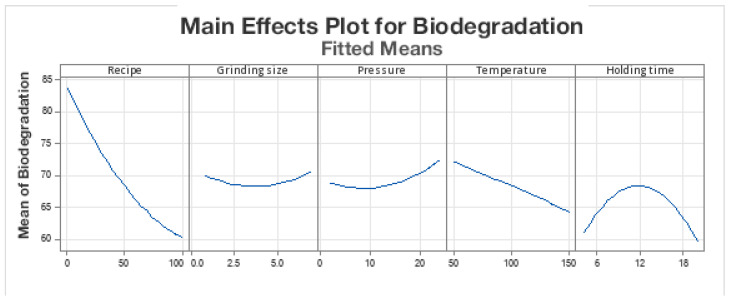
Main effects plot for biodegradation rate.

According to the biodegradation analysis, sample 8 had the highest weight loss, whereas sample 9 had the lowest one. Moreover, the degradation rate was slower for the first 30 days because soil microbial biota acclimatized to the new environment and increased after 40 days. The constituents such as cellulose, hemicellulose and lignin present in the peach and apple waste provide much-desired carbon sources for the growth and multiplication of microbes. Soil microorganisms produce extracellular hydrolytic enzymes. Enzymes such as pectinases, cellulases, and hemicellulases break down the complex organic polymers like cellulose, hemicellulose, pectins, and lignin into simpler sugars, organic acids, and other small molecules and initiate microbial biodegradation of the composites [9,54]. The biodegradation rate was affected by the recipe composition and molding temperature (*p* < 0.05, Table S5, Figure A6). High molding temperatures yield biocomposites with a higher density, reduced porosity, and lower moisture diffusion coefficients compared with those produced at lower temperatures. These microstructural and transport properties decrease water penetration and restrict microbial access, thereby slowing the biodegradation rate in soil. Another major factor is the recipe used for composite formation. The highest biodegradation was recorded in sample 8, which contained 100% dried apple waste. On the other hand, sample 9, produced from 100% peach waste, had the lowest weight loss. This could be attributed to the lignin content, which is higher in peach, resulting in greater resistance to microbial attack and slower biodegradation. Similar results were reported by Feijoo et al. (2023), who found that the lignin from wood flour slowed down the bioassimilation of PHBV/WF by limiting the access of enzymes and water to a more easily degradable cellulose and polymer matrix [55].

## 5. Conclusions

This study demonstrated that peach and apple processing residues can be effectively converted into fully biodegradable, binder-free lignocellulosic composites using hot compression molding. By systematically varying the formulation and processing parameters through a definitive screening design, clear links were established between the processing conditions, composite microstructure, and final material performance. In particular, the molding pressure and holding time played a decisive role in controlling the composite thickness and density, whereas the molding temperature significantly affected the thickness, density, moisture diffusion coefficient, flexural strength and biodegradation. The density, moisture diffusion coefficient, flexural strength and biodegradation were also influenced by the formulation, while the particle size of the raw material significantly affected both the density and moisture diffusion coefficient. Overall, the findings validate a low-cost and sustainable strategy for valorizing fruit waste into functional lignocellulosic biocomposites with a favorable mechanical behavior and rapid biodegradability. By eliminating the need for synthetic binders or polymeric matrices, this approach offers a promising alternative for the development of eco-friendly packaging, disposable foodservice products, and other single-use items. The insights gained into the structure–process–property relationships of these materials provide a foundation for optimizing industrial-scale production and for expanding the use of agricultural residues in circular bioeconomy applications.

## Figures and Tables

**Table 1:** Physicochemical properties of by-products (avg ± SD, *n* = 3).

Properties	Peach	Apple
Moisture %	4.3 ± 0.2	7.2 ± 0.3
Ash %	2.5 ± 0.1	3.2 ± 0.1
Density (g/cm^3^)	0.1 ± 0.01	0.4 ± 0.1

**Table 2:** Compositional analysis of peach and apple waste according to the NREL protocols (wt.% dry base, RSD ± 6%).

Composition	Peach	Peach EF *	Apple	Apple EF *
Glucan	20.6	30.3	17.7	33.2
Xylan	11.4	15.7	5.2	7.4
Galactan	4.2	4.6	5.5	6.1
Arabinan	0.0	0.0	0.0	0.0
Mannan	3.9	4.1	0.0	6.8
Lignin	49.8	43.7	37.4	42.5
Acid-Soluble Lignin	1.8	2.3	3.2	1.6
Acid-Insoluble Lignin	47.9	41.4	34.2	40.8
Acetyl Groups	0.2	0.0	2.1	1.0
Ash	2.5	1.9	3.2	0.5
Total **	93	100	71	97
Extractives	28.9	-	61.9	-
Extractive in H_2_O	8.5	-	45.5	-
Extractive in Ethanol	20.4	-	16.4	-

* Solid samples derived after the extraction of extractives. ** Total = Glucan + Xylan + Galactan + Arabinan + Mannan + Lignin + Acetyl units + Ash.

**Table 3:** Main physical properties of samples (avg ± SD, *n* = 3) ^1^.

Sample	Thickness (mm)	Density (g/cm^3^)	Moisture Diffusion Coefficient D_eff_ (×10^−11^ m^2^/s)
1	1.09 ± 0.01 ^def^	1.158 ± 0.018 ^c^	3.77 ± 0.08 ^fg^
2	1.03 ± 0.04 ^fg^	1.252 ± 0.019 ^b^	11.32 ± 0.19 ^d^
3	1.06 ± 0.02 ^efg^	1.162 ± 0.023 ^c^	7.69 ± 0.30 ^e^
4	1.38 ± 0.03 ^ab^	0.909 ± 0.017 ^fg^	4.90 ± 0.14 ^ef^
5	1.14 ± 0.01 ^cdef^	1.178 ± 0.021 ^c^	2.67 ± 0.09 ^fg^
6	1.18 ± 0.06 ^cde^	1.188 ± 0.017 ^bc^	15.30 ± 0.62 ^c^
7	1.02 ± 0.04 ^fg^	1.084 ± 0.026 ^d^	3.33 ± 0.04 ^fg^
8	1.24 ± 0.07 ^bcd^	1.544 ± 0.020 ^a^	44.13 ± 2.59 ^a^
9	1.04 ± 0.03 ^efg^	1.202 ± 0.025 ^bc^	2.84 ± 0.11 ^fg^
10	1.47 ± 0.04 ^a^	0.858 ± 0.007 ^g^	11.27 ± 0.48 ^d^
11	0.92 ± 0.01 ^g^	1.185 ± 0.013 ^bc^	1.54 ± 0.08 ^g^
12	1.29 ± 0.08 ^bc^	0.972 ± 0.017 ^ef^	5.28 ± 0.25 ^ef^
13	1.24 ± 0.01 ^bc^	1.017 ± 0.018 ^de^	25.91 ± 1.16 ^b^

^1^ Different superscript letters (a, b, etc.) correspond to significant differences, *p* < 0.05.

## Data Availability

Data will be made available on request.

## Supplementary Materials

The following supporting information can be downloaded at: https://www.mdpi.com/article/10.3390/biomimetics11040274/s1, Figure S1: Three point bending test in a biodegradable composite; Figure S2: Composite (a) before and (b) after three point bending test; Table S1: Analysis of Variance for thickness; Table S2: Analysis of Variance for density; Table S3: Analysis of Variance for moisture diffusion coefficient; Table S4: Analysis of Variance for flexural strength; Table S5: Analysis of Variance for biodegradation.

## Appendix A

**Table A1:** Definitive screening design for the production of composites.

A/A	Sample Number	Recipe (%)	Grinding Size (mm)	Pressure (T/m^2^)	Temperature (°C)	Holding Time (min)
1	1A	50	3.9	13	100	12
2	1B	50	3.9	13	100	12
3	2A	0	0.8	24	150	4
4	5A	100	0.8	2	150	12
5	7A	100	0.8	24	100	20
6	13A	0	7	2	100	4
7	4A	100	7	2	50	20
8	8A	0	7	24	50	12
9	7B	100	0.8	24	100	20
10	5B	100	0.8	2	150	12
11	1C	50	3.9	13	100	12
12	12A	100	3.9	24	50	4
13	6A	0	0.8	13	50	20
14	10A	50	0.8	2	50	4
15	8B	0	7	24	50	12
16	4B	100	7	2	50	20
17	12B	100	3.9	24	50	4
18	11A	50	7	24	150	20
19	6B	0	0.8	13	50	20
20	11B	50	7	24	150	20
21	4C	100	7	2	50	20
22	5C	100	0.8	2	150	12
23	12C	100	3.9	24	50	4
24	8C	0	7	24	50	12
25	13B	0	7	2	100	4
26	2B	0	0.8	24	150	4
27	10B	50	0.8	2	50	4
28	3A	0	3.9	2	150	20
29	3B	0	3.9	2	150	20
30	9A	100	7	13	150	4
31	2C	0	0.8	24	150	4
32	9B	100	7	13	150	4
33	9C	100	7	13	150	4
34	3C	0	3.9	2	150	20
35	11C	50	7	24	150	20
36	13C	0	7	2	100	4
37	10C	50	0.8	2	50	4
38	6C	0	0.8	13	50	20
39	7C	100	0.8	24	100	20
