# Supplementary material for: From Waste to Value: Fruit Biofillers in Biodegradable Composite Materials
Source: Biomimetics (Basel). 2026 Apr 15;11(4):274. doi: 10.3390/biomimetics11040274 (PMC13114007; doi:10.3390/biomimetics11040274)
Supplement: Supplementary file 1 [file biomimetics-11-00274-s001.zip › biomimetics-4240723-supplementary.pdf]

Supplementary material

Table S1: Analysis of Variance for thickness

Source	DF	Adj SS	Adj MS	F-Value	P-Value
**Model**	**11**	**0.928334**	**0.084394**	**78.59**	**0.000**
**Linear**	**5**	**0.873319**	**0.174664**	**162.64**	**0.000**
Recipe	1	0.002254	0.002254	2.10	0.160
Grinding size	1	0.000100	0.000100	0.09	0.763
Pressure	1	0.132595	0.132595	123.47	0.000
Temperature	1	0.592893	0.592893	552.09	0.000
Holding time	1	0.076759	0.076759	71.48	0.000
**Square**	**5**	**0.057206**	**0.011441**	**10.65**	**0.000**
Recipe*Recipe	1	0.000006	0.000006	0.01	0.939
Grinding size*Grinding size	1	0.000081	0.000081	0.08	0.785
Pressure*Pressure	1	0.012535	0.012535	11.67	0.002
Temperature*Temperature	1	0.012248	0.012248	11.41	0.002
Holding time*Holding time	1	0.001404	0.001404	1.31	0.264
**2-Way Interactions**	**1**	**0.000130**	**0.000130**	**0.12**	**0.731**
Recipe*Grinding size	1	0.000130	0.000130	0.12	0.731
**Error**	**24**	**0.025774**	**0.001074**		
**Pure Error**	**23**	**0.025774**	**0.001121**		
**Total**	**35**	**0.954108**			

Table S2: Analysis of Variance for density

Source	DF	Adj SS	Adj MS	F-Value	P-Value
**Model**	**11**	**1.06500**	**0.096819**	**60.56**	**0.000**
**Linear**	**5**	**0.57015**	**0.114030**	**71.33**	**0.000**
Recipe	1	0.20074	0.200737	125.56	0.000
Grinding size	1	0.02646	0.026463	16.55	0.000
Pressure	1	0.25007	0.250071	156.42	0.000
Temperature	1	0.07742	0.077419	48.43	0.000
Holding time	1	0.01546	0.015459	9.67	0.004
**Square**	**5**	**0.46711**	**0.093422**	**58.44**	**0.000**
Recipe*Recipe	1	0.06785	0.067852	42.44	0.000
Grinding size*Grinding size	1	0.02082	0.020819	13.02	0.001
Pressure*Pressure	1	0.03933	0.039331	24.60	0.000
Temperature*Temperature	1	0.04806	0.048057	30.06	0.000
Holding time*Holding time	1	0.13511	0.135108	84.51	0.000
**2-Way Interactions**	**1**	**0.01206**	**0.012059**	**7.54**	**0.011**
Recipe*Grinding size	1	0.01206	0.012059	7.54	0.011
**Error**	**27**	**0.04317**	**0.001599**		
**Pure Error**	**26**	**0.01434**	**0.000551**		
**Total**	**38**	**1.10817**			

Table S3: Analysis of Variance for moisture diffusion coefficient

**Source**	**DF**	**Adj SS**	**Adj MS**	**F-Value**	**P-Value**
**Model**	**11**	**5162.58**	**469.33**	**97.44**	**0.000**
**Linear**	**5**	**3685.26**	**737.05**	**153.02**	**0.000**
Recipe	1	2184.36	2184.36	453.51	0.000
Grinding size	1	376.59	376.59	78.19	0.000
Pressure	1	51.96	51.96	10.79	0.003
Temperature	1	901.57	901.57	187.18	0.000
Holding time	1	170.79	170.79	35.46	0.000
**Square**	**5**	**825.86**	**165.17**	**34.29**	**0.000**
Recipe*Recipe	1	272.08	272.08	56.49	0.000
Grinding size*Grinding size	1	265.38	265.38	55.10	0.000
Pressure*Pressure	1	2.76	2.76	0.57	0.455
Temperature*Temperature	1	32.10	32.10	6.66	0.016
Holding time*Holding time	1	119.40	119.40	24.79	0.000
**2-Way Interactions**	**1**	**147.39**	**147.39**	**30.60**	**0.000**
Recipe*Grinding size	1	147.39	147.39	30.60	0.000
**Error**	**27**	**130.05**	**4.82**		
**Pure Error**	**26**	**26.70**	**1.03**		
**Total**	**38**	**5292.63**			

Table S4: Analysis of Variance for flexural strength

Source	DF	Adj SS	Adj MS	F-Value	P-Value
**Model**	**11**	**507.520**	**46.138**	**14.72**	**0.000**
**Linear**	**5**	**393.044**	**78.609**	**25.07**	**0.000**
Recipe	1	85.765	85.765	27.36	0.000
Grinding size	1	1.023	1.023	0.33	0.575
Pressure	1	12.826	12.826	4.09	0.057
Temperature	1	267.293	267.293	85.25	0.000
Holding time	1	15.680	15.680	5.00	0.038
**Square**	**5**	**71.730**	**14.346**	**4.58**	**0.007**
Recipe*Recipe	1	40.516	40.516	12.92	0.002
Grinding size*Grinding size	1	1.481	1.481	0.47	0.500
Pressure*Pressure	1	8.319	8.319	2.65	0.120
Temperature*Temperature	1	22.053	22.053	7.03	0.016
Holding time*Holding time	1	0.423	0.423	0.14	0.717
**2-Way Interactions**	**1**	**13.103**	**13.103**	**4.18**	**0.055**
Recipe*Grinding size	1	13.103	13.103	4.18	0.055
**Error**	**19**	**59.570**	**3.135**		
**Pure Error**	**18**	**32.567**	**1.809**		
**Total**	**30**	**567.090**			

Table S5: Analysis of Variance for biodegradation

Source	DF	Adj SS	Adj MS	F-Value	P-Value
**Model**	**11**	**1672.14**	**152.01**	**2176.13**	**0.017**
**Linear**	**5**	**1554.87**	**310.97**	**4451.75**	**0.011**
Recipe	1	1369.98	1369.98	19611.86	0.005
Grinding size	1	0.74	0.74	10.56	0.190
Pressure	1	28.94	28.94	414.34	0.031
Temperature	1	150.12	150.12	2149.02	0.014
Holding time	1	5.10	5.10	72.97	0.074
**Square**	**5**	**116.23**	**23.25**	**332.77**	**0.042**
Recipe*Recipe	1	28.80	28.80	412.22	0.031
Grinding size*Grinding size	1	8.38	8.38	119.99	0.058
Pressure*Pressure	1	5.09	5.09	72.93	0.074
Temperature*Temperature	1	0.02	0.02	0.34	0.662
Holding time*Holding time	1	74.30	74.30	1063.62	0.020
**2-Way Interactions**	**1**	**18.72**	**18.72**	**268.05**	**0.039**
Recipe*Grinding size	1	18.72	18.72	268.05	0.039
**Error**	**1**	**0.07**	**0.07**		
**Total**	**12**	**1672.21**			

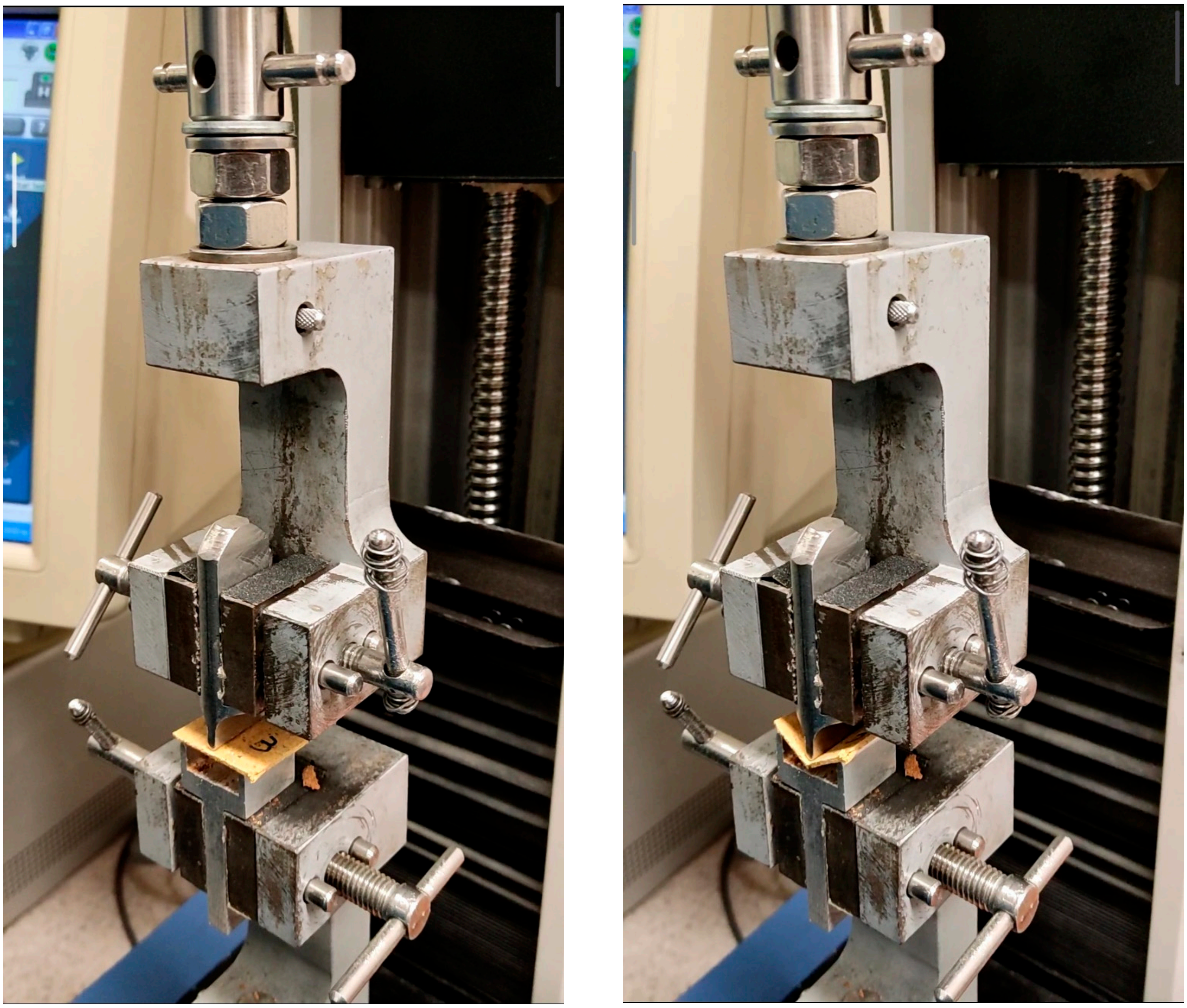

Figure S1: Three point bending test in a biodegradable composite.

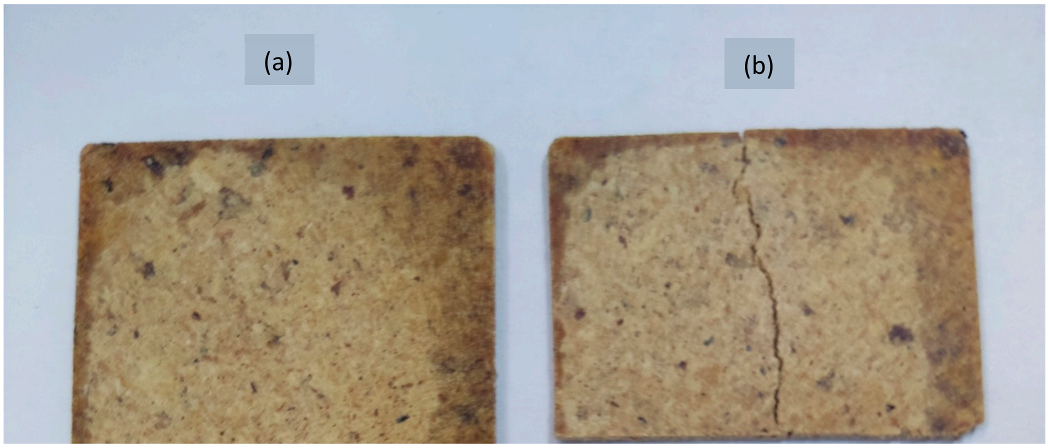

Figure S2: Composite (a) before and (b) after three point bending test.
